# Effect of Phone Text Message Reminders on Compliance with Rabies Post-Exposure Prophylaxis following Dog Bites in Rural Kenya

**DOI:** 10.3390/vaccines11061112

**Published:** 2023-06-18

**Authors:** Veronicah M. Chuchu, Nyamai Mutono, Philet Bichanga, Philip M. Kitala, Daniel Ksee, Mathew Muturi, Athman Mwatondo, Carolyne Nasimiyu, Lawrence Akunga, Amine Amiche, Katie Hampson, Samuel M. Thumbi

**Affiliations:** 1 Centre for Global Health Research, Kenya Medical Research Institute, Kisumu 1578-40100, Kenya; thumbi.mwangi@wsu.edu; 2 Department of Public Health, Pharmacology and Toxicology, University of Nairobi, Nairobi 29053-00625, Kenya; pkitala@uonbi.ac.ke; 3 Paul G. Allen School for Global Health, Washington State University, Pullman, WA 99164-7090, USAcarolyne.nasimiyu@wsu.edu (C.N.); 4 Centre for Epidemiological Modelling and Analysis, University of Nairobi, Nairobi 19676-00202, Kenya; 5 Department of Health Services, Government of Makueni County, Makueni 95-90300, Kenya; 6 Department of Agriculture, Irrigation, Livestock and Fisheries Development, Government of Makueni County, Makueni 78-90300, Kenya; drdmksee@gmail.com; 7 Zoonotic Disease Unit, Joint One Health Office of the Ministry of Health and the Ministry of Agriculture, Livestock and Fisheries, Government of Kenya, Nairobi 20811-00202, Kenya; muturimathew@gmail.com (M.M.); amwatondo@yahoo.com (A.M.); 8 Sanofi-Aventis Kenya Limited, Nairobi 20337-00200, Kenya; lawrence.akunga2@sanofi.com; 9 Sanofi, Dubai 53899, United Arab Emirates; amine.amiche@sanofi.com; 10 Institute of Biodiversity, Animal Health & Comparative Medicine, Graham Kerr Building, University of Glasgow, Glasgow G12 8QQ, UK; katie.hampson@glasgow.ac.uk; 11 Institute of Immunology and Infection Research, School of Biological Sciences, University of Edinburgh, Edinburgh EH8 9YL, UK

**Keywords:** rabies, SMS, PEP, regimen

## Abstract

The prompt administration of post-exposure prophylaxis (PEP) is one of the key strategies for ending human deaths from rabies. A delay in seeking the first dose of rabies PEP, or failure to complete the recommended dosage, may result in clinical rabies and death. We assessed the efficacy of short message system (SMS) phone texts in improving the adherence to scheduled PEP doses among bite patients in rural eastern Kenya. We conducted a single-arm, before-after field trial that compared adherence among bite patients presenting at Makueni Referral Hospital between October and December 2018 (control) and between January and March 2019 (intervention). Data on their demographics, socio-economic status, circumstances surrounding the bite, and expenditures related to the bite were collected. A total of 186 bite patients were enrolled, with 82 (44%) in the intervention group, and 104 (56%) in the control group. The odds of PEP completion were three times (OR 3.37, 95% CI 1.28, 10.20) more likely among patients who received the SMS reminder, compared to the control. The intervention group had better compliance on the scheduled doses 2 to 5, with a mean deviation of 0.18 days compared to 0.79 days for the control group (*p* = 0.004). The main reasons for non-compliance included lack of funds (30%), and forgetfulness (23%) on days for follow-up treatment, among others. Nearly all (96%, *n* = 179) the bite patients incurred indirect transport costs, at an average of USD 4 (USD 0–45) per visit. This study suggests that the integration of SMS reminders into healthcare service delivery increases compliance with PEP, and may strengthen rabies control and elimination strategies.

## 1. Introduction

Rabies, a fatal viral disease transmitted to humans mainly by domestic dogs, is a neglected zoonosis that primarily affects underserved populations that have limited access to healthcare. Every year, rabies is estimated to kill 59,000 people globally, mostly children 15 years and below in Africa and Asia [1,2], despite the development of effective vaccines against rabies in humans, and in dogs [3]. Although rabies is always fatal once clinical signs manifest, the disease is preventable with timely treatment after exposure to the rabies virus. The World Health Organization (WHO) recommendations for bite patients are the immediate thorough cleaning of the wound with soap and water or virucidal agents for approximately 15 min, followed by the administration of an anti-rabies vaccine and, where there are multiple severe bites, particularly to the head and upper trunk, the infiltration of rabies immunoglobulin into and around the wound(s) [4].

In developing countries, including Kenya, access to post-exposure vaccines is poor due to its unaffordable cost and unavailability [2]. The lack of access to post-exposure prophylaxis (PEP); or deviations from the WHO recommendations, such as delays in seeking PEP, and incomplete courses of PEP; increases the risk of clinical rabies and death [5,6]. To increase access and availability, the WHO has updated their recommendations for rabies post-exposure vaccination regimens, from the 0.5 mL or 1 mL per dose of intramuscular (IM) Essen regimen schedule of five doses on day 0, 3, 7, 14, and 28 (5-dose Essen regimen); to either a one-week intradermal (ID) schedule that is dose-sparing and cost-effective, which consists of 0.1 mL of vaccine per dose, given as two ID injections on day 0, 3, and 7; or, a two-week, intramuscular (IM)-injection, 4-dose post-exposure-prophylaxis regimen, with injections on day 0, 3, 7, and between days 14 and 28 (4-dose Essen regimen) [4,5]. However, the ID regimen is only partially implemented in many rabies-endemic countries, including Kenya, which is still following the 5-dose IM Essen regimen.

In Kenya, rabies is endemic across the country, and has been estimated to cause over 500 (95% CI 134, 1100) deaths annually [7]. A national strategic plan for the elimination of dog-mediated human rabies by 2030 was developed and adopted in 2014 [7]. The key components of the plan include mass dog vaccination, timely provision of pre- and post-exposure vaccines, enhanced rabies surveillance for both human and animal populations, and public health education and awareness on rabies, and its prevention and control [7]. Additionally, the elimination activities would be phased, starting with pilot counties selected for their high burden of rabies, before a scale-up to the rest of the country. Makueni County, where this study was conducted, is one of the five counties selected as pilot areas for Kenya’s rabies elimination strategy [8]. Previously, we reported large variability in the availability of rabies post-exposure vaccines in the country, with pilot counties having shorter stockout periods [9]. Information and medical products, vaccines and technologies, and their access and uptake form two of the six pillars of the WHO Health System Building Blocks. The WHO encourages the strengthening of health systems to achieve the health goals set out [10]. Innovations, such as the use of mobile phones, can play a part. The use of mobile phone applications in health has increased globally, due to their availability and ability to deliver scalable interventions [11]. Our previous studies showed the role that mobile phones can play in improving the detection of outbreaks of zoonotic diseases in the country [12,13]. The use of text message reminders has been shown to improve patient compliance with, for example, childhood immunization attendance, and appointment reminders, across different geographical settings and healthcare services [14,15,16]. The use of short message systems (SMSs) has further been reported to improve dog owners’ participation in mass dog vaccinations in Haiti [17], and compliance to PEP regimens in Tanzania [18]. In this study, our objective was to assess the effect of SMS reminders on compliance with the five-dose Essen rabies PEP regimen, and the factors associated with compliance among dog bite patients in Makueni County.

## 2. Materials and Methods

### 2.1. Study Area

**Figure 1:**
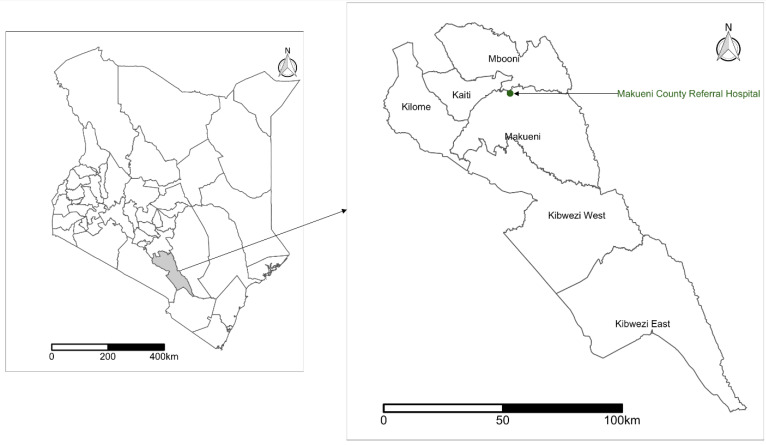
Map of Kenya, highlighting the location of Makueni County (**left**) and the Makueni County Referral Hospital in Makueni Subcounty (**right**). Shapefile source: Database of Global Administrative Areas [21].

The study was conducted in Makueni County, one of the five counties selected as pilot areas for the implementation of the Kenya rabies elimination strategy. Makueni County is divided into six sub-counties (Mbooni, Kaiti, Makueni, Kibwezi East, Kibwezi West, and Kilome) (Figure 1), and has an estimated human population of 987,653 as of 2019 [19]. The main referral hospital for the county is based in Wote town, which serves as the administrative center for the county. Makueni County has a total of 248 public health facilities. Of these, eleven are sub-county health facilities, and one is a county-referral hospital. The twelve (sub-county and county-referral) health facilities are mandated to provide anti-rabies vaccines to the community. However, other private health facilities provide the vaccines.

The cost of anti-rabies vaccines varies. In public health facilities, the cost of the vaccine ranges from USD 0 per dose; for patients under the Makueni universal health program, who are enrolled by paying an enrollment fee of USD 5; to USD 8.5 per dose, for patients who are not under the universal health program. In private health facilities, the cost of one dose may range from USD 10 to USD 25. The estimated annual bite incidence for Makueni County is 342 cases per 100,000 people per year [20].

### 2.2. Study Design and Sample Size Calculation

We conducted a single-arm, before-after field trial among dog bite patients presenting at the Makueni County Referral Hospital between October 2018 and March 2019. The study participants were allocated to either the control or the treatment group, based on the time of their recruitment. Data on the bite patient’s name, their contacts, details of their next of kin, the site of the bite, the species of the biting animal, the bite severity, the vaccination status of the biting animal, and the date of each PEP dose received were extracted from the anti-rabies vaccine register at the County Referral Hospital. Beginning in January 2019, an SMS reminder written in both English and the local dialect, Kamba, was sent out to all bite patients a day before their next dose of PEP. The SMS reminder was sent a day before each dose, until the scheduled date of the last dose of PEP. Bite patients recruited into the study between October and December 2018 were designated as the control group. As is routine, this group received a medical card indicating the return date for the subsequent dose. Those recruited from January to March 2019 were designated as the intervention group. This group received the medical card, and SMS reminders.

**Figure 2:**
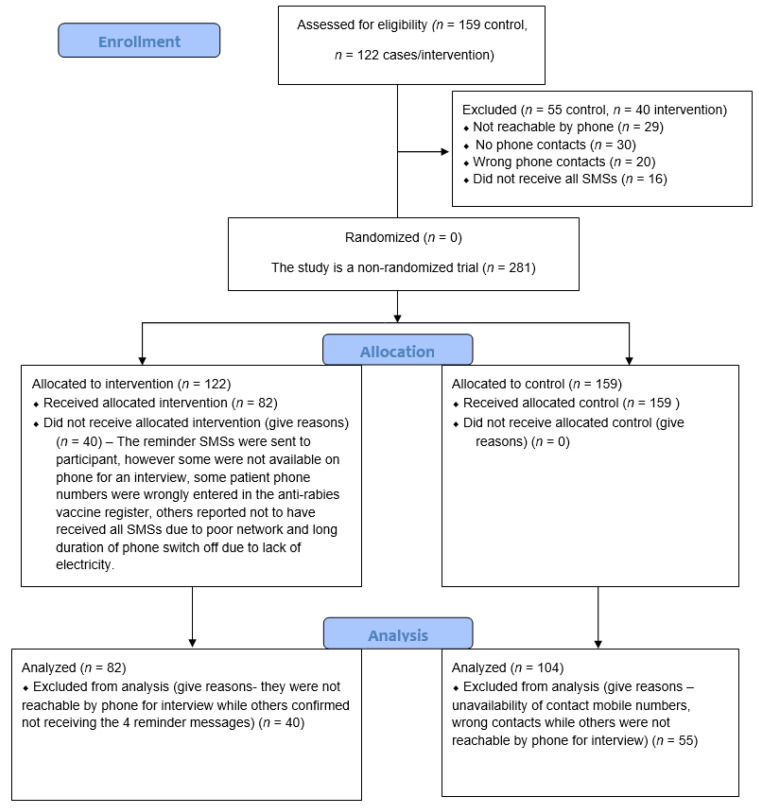
CONSORT flow diagram displaying the progress of all participants throughout the trial.

To collect data on other factors affecting PEP completion and adherence, a phone interview was conducted with both groups in May, June, and July 2019, with the majority (76%) being contacted between May and June 2019. In the control group, all bite patients who responded to the phone interview were enrolled in the study, while in the intervention group, all bite patients who responded to the interview and were confirmed to have received all four SMS reminders were considered for the final data analysis (Figure 2).

The bite case data from the referral hospital that were available before the start of the study showed an average of 30 bite cases per month. To establish the number of human dog bite cases to be recruited to either the control or the intervention group, we hypothesized that SMS reminders would increase compliance, and calculated the required sample size to detect a 20% increase in compliance in the group receiving SMS reminders compared to the group not receiving the reminders, with 80% power, and at the significance level of 0.05. We estimated a sample size of 90 bite patients was required for each of the control and intervention groups, assuming an increase in compliance from 60% to 80% following the introduction of the SMS reminder. The power calculations were carried out using the “pwr” package [22] of the R statistical computing software version 3.5.1 [23]. However, all dog bite patients recorded in the register during the study period and in possession of a phone were enrolled in the study, to cater for withdrawals. The primary outcome measures were the number of participants that completed the five-dose Essen rabies vaccine regimen in the control versus the intervention group, and the number of participants adhering to the scheduled date of the five-dose Essen vaccine regimen in the two groups.

### 2.3. Data Collection

For each study participant, a questionnaire was administered by phone call at the end of the PEP period, after they had provided verbal consent to participate in the study. For bite patients below 18 years, a parent or guardian was interviewed. The data collected included the demographics of the bite patient (age, gender), their home location, the characteristics of the bite, their PEP compliance (the date of the bite, and date of the subsequent PEP injections), and data on putative factors affecting PEP completion, which included the SMS reminder, ownership of health insurance cover, the education level of the bite patient, dog ownership status, the vaccination status of the biting animal, the fate of the biting dog (alive, dead, or disappeared), the time taken to reach the health facility, the means of transport used, the total transport cost, whether the bite patient was accompanied to the health facility, the total cost of the PEP, the source of money to cover the incurred cost, the household head occupation and age, the total monthly household income, the number of people in the household, and their livestock ownership status.

### 2.4. Data Analysis

The study questionnaires were programmed in the CommCare^®^ data collection tool, to allow for electronic data capture using mobile phones. Data were then downloaded as a Microsoft Excel file, and statistical analysis was undertaken, using the R computing language [23]. To assess the factors associated with the completion of the five doses of PEP, the uptake of PEP was dichotomously classified based on whether the patient had completed the full regimen or not (1 or 0), and univariate analysis was carried out on the different independent variables (Chi-square tests and t-tests, for categorical and numerical variables, respectively).

To understand the factors associated with the compliance with, and completion of, the anti-rabies vaccine schedule, multivariable logistic regressions were conducted for the compliance with each dose. The Akaike information criterion backward and forward stepwise algorithm was used to identify the suitable factors for each model.

### 2.5. Clinical Trial

The study trial is registered at US National Institute of Health (clinicalTrial.gov) accessed on 28 April 2022, identifier number NCT05350735. https://clinicaltrials.gov/ct2/show/NCT05350735 posted on 28 April 2022. CONSORT 2010 checklist reporting this non-randomized trial is available as Supplementary File S1 and clinical trial protocol as Supplementary File S2. 

## 3. Results

### 3.1. Demographic and Socio-Economic Characteristics

A total of 281 bite patients were recruited in the study, 159 (57%) in the control group, and 122 (43%) in the intervention group. About a third (*n* = 55, 35%) of the control group were excluded from the study, due to unavailability of contact mobile numbers, or wrong phone numbers, while others were not reachable by phone for the interview. In the intervention group, 40 (33%) bite patients were excluded from the study. Of these 40, 16 (40%) did not receive all the reminders due to poor phone network, or phones being off for long periods, due to a lack of electricity to charge them. The rest were excluded due to their unavailability for interview by phone, and wrong contact numbers.

**Figure 3:**
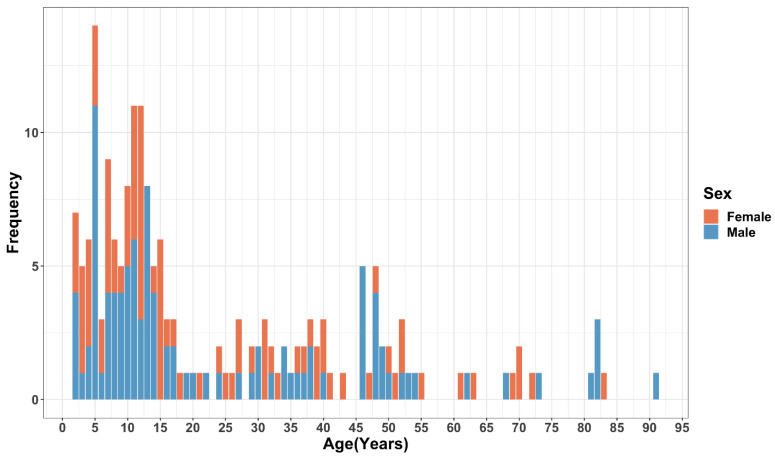
Age distribution of the bite patients in years and by gender.

A total of 186 bite patients were considered for analysis, 104 (56%) in the control group, and 82 (44%) in the intervention group. During the follow-up period, none of the patients were recorded dead due to rabies. More than half (*n* = 101, 54%) of the bite patients were male, and the median age of the bite patients was 14 years (IQR 8, 38 years) (Table 1). The majority (*n* = 104, 56%) of the bite patients were children below the age of 15 years (Figure 3). Half (*n* = 93) of the patients were in primary school, followed by 50 (27%) and 39 (21%) in secondary and tertiary school, respectively. The majority (*n* = 173, 93%) of the bite patients resided in rural areas of Makueni County. The average household size of the bite patients was six people (range 1–32). The main occupation for most households was farming, followed by business. Nearly two-thirds (*n* = 123, 66%) of the households had a monthly income of less than USD 100 (Table 1).

In total, 141 bite patients (76%) used public transportation to reach the health facilities. The average cost of transportation spent by the bite patients was USD 4 per visit. The average time taken to reach the health facility was 47 min (range 2–240 min). Seventy percent (*n* = 130) of the bite patients were accompanied to the health facility at some point during the treatment period, while 2% (*n* = 4) of the patients sought accommodation while attending the health facility. Almost all (*n* = 174, 94%) of the bite patients were beneficiaries of a health insurance scheme which reduced the cost of treatment at the health facility (Table 1).

### 3.2. Characteristics of the Bite and Biting Animal

The most common bite sites were the legs (*n* = 110, 59%) and the arms (*n* = 56, 30%). Most of the bites were classified as either category two (*n* = 88, 47%) or category three (*n* = 81, 44%), as per the WHO categorization of bite wounds [5]. More than half (*n* = 109, 59%) of the patients were bitten by dogs known to them, but not their own dogs, whereas nearly a third (*n* = 57, 31%) were bitten by their own dogs. Only 43% of the biting dogs (*n* = 80) had a history of vaccination, while 69% (*n* = 128) were alive, and 15% (*n* = 27) dead at the time of the interview (Table 2).

### 3.3. Effect of SMS Reminders on Compliance with the PEP Regimen

Among the 104 participants recruited into the control group, 81 (78%) completed the five doses of the PEP vaccine. Of the 82 participants in the intervention group, 76 (93%) completed the PEP doses, resulting in a 15% increase in completion rates. Out of the 29 (16%) bite patients who did not complete the five doses of PEP, 23 (*n* = 79%) were in the control period.

The odds of PEP completion with SMS reminders were three times (OR 3.37, 95% CI 1.28, 10.20) more likely in the intervention group compared to the participants in the control group (Table 3).

**Figure 4:**
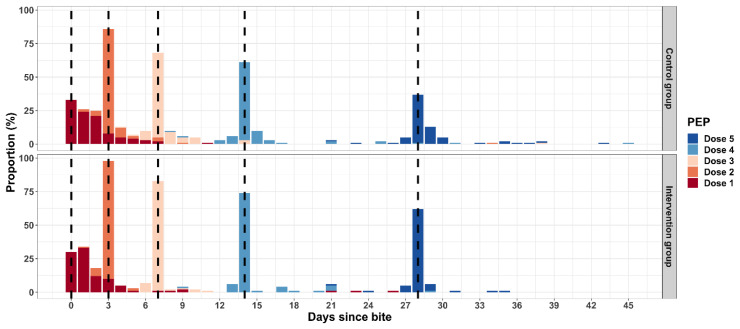
Figure showing the timing of each of the five doses of PEP, per the Essen regimen, for the control and intervention groups. The dashed lines highlight the WHO-recommended Essen schedule for the five doses of PEP for bite cases [5].

We studied the compliance with the day of PEP administration for each of the five doses against the WHO recommendation for the Essen regimen for days 0, 3, 7, 14, and 28. Nearly a third (*n* = 59, 32%) of the bite patients received the first dose of PEP less than 24 h after the bite, while 45% took the dose one to two days after the bite. Compliance with the scheduled date of PEP improved to more than 70% receiving second, third, fourth, and fifth dose on day 3, 7, 14, and 28 after the bite, respectively. The average time from the bite to the first dose of PEP was 1.99 days, with no statistical differences between the control and intervention groups. However, we found that the intervention group had better compliance on the scheduled doses 2 to 5, with a mean deviation of 0.18 days, compared to 0.79 days for the control group (*p* = 0.004) (Figure 4).

### 3.4. Factors Affecting PEP Completion and Compliance

**Figure 5:**
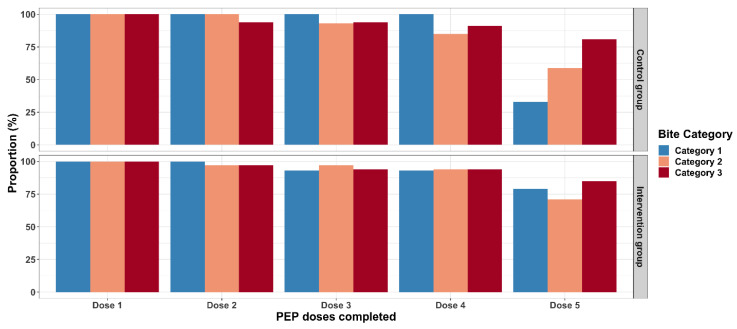
Figure showing the proportion of bite patients who completed the different PEP doses per bite category, in the intervention and control groups.

Our visualization of the proportion of PEP doses completed per bite category showed that dose two to four had a high uptake, of more than 80%. The comparison of PEP dose completion between the intervention and control groups showed that the intervention group had a relatively higher PEP uptake, of more than 70% in all the bite categories, while patients in the control group with bite category III had a higher rate of completion of the five doses. The drop-out rate between vaccinations was higher in the control group, especially at the 5th injection, where 16 bite patients dropped out (Figure 5).

In addition, the odds of male patients completing treatment was three times more likely compared to female bite patients (OR 2.95, CI 1.24–7.39). The cost of transport to the health facility, monthly family income, the age of the bite patient, the ownership status and fate of the biting dog, the bite category, and the home location were not significantly associated with the completion of PEP among the bite patients (Table 3).

Family income per month was significantly associated with the uptake of the first dose less than 24 h after the bite, where the odds were three times (OR 3.2, CI 1.645–6.174) more likely for households with monthly earnings of >USD100.

The odds of compliance with the second dose of PEP, due three days after the bite, were four times (OR 4, CI 1.33–15.31) more likely in the intervention group, compared to the control group. This was similar to the uptake from the third to the fifth dose, where patients who had been sent SMS reminders had a higher likelihood of complying with the recommended PEP scheduled date (Table 3). Bite patients with category III bites had a higher likelihood of receiving PEP dose 3, 4, and 5 on the recommended scheduled date. In addition, compared to females, male patients had a significantly lower likelihood of PEP compliance during the third (OR 0.3, CI 0.09–0.69) and fourth (OR 0.36, CI 0.15–0.82) dose (Table 3).

A total of 128 (69%) patients were bitten by dogs that were still alive at the time of the interview. From this group of patients, 82% (105/128) completed the five doses.

### 3.5. Reasons for Non-Compliance and PEP Cost

Among the 29 (16%) patients who did not complete the five doses, the main reasons for non-compliance included lack of funds (*n* = 9, 30%), forgetfulness (*n* = 7, 23%) on days for follow-up treatment, unavailability of PEP in the facility (*n* = 5, 17%), or that the biting dog was still alive (*n* = 5, 17%), among others (Table 4). However, none of the bite patients in the intervention group failed to complete the five doses due to forgetfulness, while only 22% (*n* = 2/9) who had mentioned lack of funds were in the intervention group. Of those who had mentioned PEP unavailability, and that the biting dog was still alive, 60% (*n*= 3/5) and 40% (*n*= 2/5) were in the intervention group, respectively (Table 4).

The majority (89%) of the bite patients did not purchase the anti-rabies vaccine, since the vaccine was available at the hospital under the Universal Health Coverage program run by the county, whereby healthcare services are free for all households that enroll into the program by paying the USD 5 enrollment fee. Participants who reported incurring costs on PEP spent a minimum of USD 5 and a maximum of USD 45. Nearly all (96%, *n* = 179) of the bite cases incurred transport costs, with an average of USD 4.6 (range 0–45 USD). More than two-thirds of the bite patients (70%) were accompanied to the health facility, resulting in lost earnings and missed school time for school-aged children. In total, 61 (75%) bite cases reported using their own savings to cover treatment costs, while 18 (22%) reported selling livestock (mostly goats and chickens) to cover treatment costs (Table 4).

## 4. Discussion

Here we report on the effect of SMS reminders in increasing proportions of PEP completion among dog bite patients, and adherence to the scheduled dates of PEP. We note that SMS reminders were associated with an increased likelihood of completing the PEP doses, compared to participants who did not receive SMS reminders. The majority of the patients who did not complete the five doses were in the control group, and cited lack of funds and forgetfulness as the main reasons for not completing the doses. In the intervention group, the main reported reasons for non-completion of the schedule were lack of access to the PEP, lack of funds, and that the biting dog was still alive. The odds of male patients completing treatment were three times more likely compared to female bite patients. The age of the bite patient, the cost of the transport to the health facility, the monthly family income, the ownership status and fate of the biting dog, the bite category, and the home location were not significantly associated with the completion of the PEP among the bite patients. The intervention group had better compliance with the scheduled doses 2 to 5, with a mean deviation of 0.18 days, compared to 0.79 days for the control group. The majority of the bite patients did not purchase the anti-rabies vaccine, since the vaccine was available for free at the hospital under the Universal Health Coverage program. Participants that reported incurring cost on PEP spent a minimum of USD 5 and a maximum of USD 45. On average, transportation costs spent by bite patients were USD 4 per visit.

Rabies is a vaccine-preventable disease in both humans and animals, by annual vaccination of 70% of the dog population to effectively control and eliminate rabies, and through prompt administration of PEP to bite patients [2,3,24,25]. Unfortunately, in many countries where rabies is endemic, PEP is unavailable, and the cost of vaccine remains high for both individuals and the government, which negatively impacts the ability of affected countries to meet the Sustainable Development Goals, especially the goal to eradicate extreme poverty and hunger and improve health by 2030 [26,27,28]. The communities in these endemic countries also remain insufficiently aware of rabies risks and prevention measures. Improving the completion of, and compliance with, the PEP regimen is critical. Studies have shown that lack of early and adequate post-exposure vaccination is the most important cause of mortality due to rabies [29,30] with reasons for non-compliance falling under lack of wages, forgotten dates, and costs incurred toward treatment [31]. With rabies being a fatal disease, a discontinuation of PEP in an endemic region where dogs are left to roam freely, without a confirmation by a trained professional on the health status of the biting animal, would expose bite patients to rabies. To ensure the judicious use of rabies vaccines, healthcare workers should undertake risk assessments to determine the need for PEP, and when to discontinue PEP. The WHO has provided guidelines on rabies pre-exposure and PEP vaccination strategies, recommending that countries switch to intradermal injections, and discontinue PEP, if the biting animal is confirmed to be negative for rabies using appropriate laboratory tests, or if the biting animal remains healthy for more than 10 days after the bite [5].

This study highlights the population that is at risk of contracting rabies as being children below the age of 15, likely due to their close relationship with dogs. Increasing patient awareness on the risks of contracting diseases, the use of SMS reminders, and medication subsidies have all been shown to increase compliance with medication [32,33]. However, despite the availability of the Universal Health Coverage program, which covers treatment costs for all members of a household after registration at USD 5, some of the bite patients, especially those who did not receive reminders, did not complete the five doses of PEP, or adhere to the timeliness of each dose of the vaccine. Reasons cited by the bite patients for not completing PEP were forgetfulness and lack of funds. This could be attributed to the inhibitive transport cost to the health facility, and the cost of PEP for patients not in the Universal Health Coverage program. These results are in accordance with what has been reported for rural areas in Africa where the incidence of rabies is high, and the residents are poor and living below the poverty line, and have limited access to healthcare [1,2]. In addition to SMS reminders, medication subsidies can be used as a synergistic strategy for increasing compliance.

For patients with bites from suspected rabid dogs, timely access to rabies vaccines, and compliance with the full course of the five doses of PEP, as recommended by the WHO, significantly reduces the risk of developing rabies, and death. Delays in seeking PEP, and incomplete courses, expose bite patients to rabies [6,24]. Here, we see delays in receiving the first dose after the bite, and lack of adherence to timeliness on the scheduled days of the subsequent doses of PEP. The delay in seeking PEP, and lack of adherence to timeliness of PEP, suggest a lack of awareness among the community of the need to seek PEP immediately after a bite, and to adhere to timeliness for the subsequent doses. This study reveals the potential of SMS reminders to improve the adherence to PEP schedules as recommended by the WHO. The bite patients who received the SMS reminders displayed a higher likelihood of complying with the timeliness of the subsequent doses after the initial dose, potentially due to the SMS reminders both reminding the patient, and reinforcing their awareness about the need for complete and timely PEP.

As many governments express interest in mobile health as a complementary strategy for strengthening health systems and achieving the health-related Sustainable Development Goals in low- and middle-income countries, the potential of mobile phone technology for improving health has been shown in different health sectors [11]. This includes the use of mobile phones in rabies surveillance and the demonstration of PEP demand in health facilities [34]. In the delivery of health interventions, SMSs have been shown to increase the completion of the five doses of PEP (irrespective of the age or location of the bite patient) [18], and to improve health outcomes, rates of post-treatment hospital return, and adherence to healthy diets and medication, especially in HIV/AIDS and diabetes patients, with up to 100% effectiveness [14,18,35,36,37]. In our study, the odds of completion of the five doses was 3.4 times more likely in patients who received the SMS reminder, compared to those who did not receive the SMS reminder. The effect of the SMS reminder was irrespective of the age of the bitten patient, the age of the household head, the fate of the biting dog, the occupation of the bite patient/next of kin, the category of bite, the vaccination status of the biting dog, the education level, ownership of health cover, the family monthly income, their livestock ownership status, the transport cost, the distance to the health facility, the source of the money used for the treatment, and the home location (urban/rural) of the bite patient.

Although the study shows significantly improved completion rates of PEP among the bite patients who received SMS reminders, the study had a limitation, in that data were collected at different times of the year. This could result in a bias in compliance with PEP, as the time and availability of resource may have not been favorable at that specific time. The design of the study also lacked the randomization of the patients into control and intervention group due to ethical consideration. The use of the SMS reminder strategy may also not be effective for older patients with lower literacy levels or lower socioeconomic status, or for communities without the power to purchase a phone, and in rural areas where network signals and connection are poor. Excluding the bite patients who were not reached by phone for interview could also introduce a potential bias, as those who received the SMSs and did not comply may be less likely to want to be interviewed.

## 5. Conclusions

Rabies continues to pose a significant public health concern in Kenya. The prevention of clinical cases of human rabies following exposure is dependent on prompt access to, and compliance with, PEP regimens. This study highlights the potential value of text messages in the delivery of public health interventions, as a complementary strategy for strengthening health systems. The integration of SMS reminders for the next dose of PEP to dog bite patients at risk of contracting rabies should work synergistically with efforts to control and eliminate rabies in endemic countries, including the provision of PEP supported by Gavi, the Vaccine Alliance. To minimize the challenges of message delivery, a protocol should be developed in hospitals to ensure the proper recording of a patient’s phone number, and confirmation of its functionality. Improved phone network signals and coverage may also increase the pool of bite patients receiving the reminders. A high level of community awareness of the need to seek PEP immediately after a bite, and to adhere to the PEP schedule, is critical, to reduce the number of human rabies cases and achieve the “Zero by 30” goal.

## Figures and Tables

**Table 1:** Demographics of the bite patients.

	Intervention Group (*n* = 82)	Control Group (*n* = 104)	Total (*N* = 186)	*p* Value
*Gender*				0.053
Female	44 (54%)	41 (39%)	85 (46%)	
Male	38 (46%)	63 (61%)	101 (54%)	
*Age*				0.688
Median	15	12	14	
Interquartile range	8–38	8–33	8–38	
*Level of education*				0.135
Non formal education	0 (0%)	4 (4%)	4 (2%)	
Primary school	47 (57%)	46 (44%)	93 (50%)	
Secondary school	20 (24%)	30 (29%)	50 (27%)	
Tertiary school	15 (18%)	24 (23%)	39 (21%)	
*Household size*				0.317
Mean (SD)	6 (4)	6 (3)	6 (4)	
Range	2–30	1–32	1–32	
*Home location*				0.316
Rural	78 (95%)	95 (91%)	173 (93%)	
Urban	4 (5%)	9 (9%)	13 (7%)	
*Age of household head*				0.684
Median	44	45	44	
Interquartile range	38–50	38–53	38–50	
Occupation ^†^				0.760
Businessperson	16 (20%)	23 (22%)	39 (21%)	
Casually employed	8 (10%)	14 (14%)	22 (12%)	
Farmer	41 (50%)	57 (55%)	98 (53%)	
Formally employed	15 (18%)	12 (12%)	27 (15%)	
Unemployed	5 (6%)	7 (7%)	12 (7%)	
*Family income per month*				0.809
≤USD 100	55 (67%)	68 (65%)	123 (66%)	
>USD 100	27 (33%)	36 (35%)	63 (34%)	
*Household ownership of livestock* ^†^				0.610
Chicken	28 (34%)	50 (48%)	78 (42%)	
Cow	38 (46.3%)	51 (49%)	89 (48%)	
Goat	46 (56.1%)	67 (64%)	113 (61%)	
Sheep	5 (6.1%)	7 (7%)	12 (7%)	
Donkey	13 (15.9%)	17 (16%)	30 (16%)	
*Means of transport to health facility* ^†^				0.366
Motorbike	28 (34%)	52 (50%)	80 (43%)	
Private vehicle	3 (4%)	1 (1%)	4 (2%)	
Public vehicle transport	58 (71%)	84 (81%)	141 (76%)	
Walking	5 (6%)	6 (6%)	11 (6%)	
*Time taken to health facility (mins)*				0.715
Median	35	30	30	
Interquartile range	30–60	30–60	30–60	
*Cost of transport (USD)*				0.671
Mean (SD)	4 (5)	4(3)	4(4)	
Range	0–42	0–17	0–42	
*Whether patient was accompanied at some point to the health facility*				0.457
No	27 (33%)	29 (28%)	56 (30%)	
Yes	55 (67%)	75 (72%)	130 (70%)	
*Whether the patient sought accommodation while attending the health facility*				0.810
No	80 (98%)	102 (98%)	182 (98%)	
Yes	2 (2%)	2 (2%)	4 (2%)	
*Health insurance used*				0.041
None	7 (9%)	5 (5%)	12 (6%)	
National Health Insurance Fund	9 (11%)	3 (3%)	12 (6%)	
Universal Health Coverage	66 (80%)	96 (92%)	162 (87%)	

^†^ Participants in more than one category.

**Table 2:** Characteristics of bites and the status of the biting animal.

Parameters	Treatment Group (*n* = 82)	Control Group (*n* = 104)	Total (*N* = 186)	*p* Value
*Bite site*				
Head/Neck	4 (5%)	5 (5%)	9 (5%)	0.982
Arms/Hands	29 (35%)	27 (26%)	56 (30%)	0.165
Trunk	1 (1%)	5 (5%)	6 (3%)	0.169
Legs	49 (60%)	61 (59%)	110 (59%)	0.879
Feet	3 (4%)	7 (7%)	10 (5%)	0.356
*Bite category*				0.004
Category 1	14 (17%)	3 (3%)	17 (9%)	
Category 2	34 (42%)	54 (52%)	88 (47%)	
Category 3	34 (42%)	47 (45%)	81 (44%)	
*Dog ownership status*				0.298
Known	47 (57%)	62 (60%)	109 (59%)	
Own	23 (28%)	34 (33%)	57 (31%)	
Unknown	12 (15%)	8 (8%)	20 (11%)	
*Vaccination status of biting animal*				0.399
Don’t know	16 (20%)	15 (14%)	31 (17%)	
Unvaccinated	35 (43%)	40 (39%)	75 (40%)	
Vaccinated	31 (38%)	49 (47%)	80 (43%)	
*Fate of biting animal*				0.319
Alive	58 (71%)	70 (67%)	128 (69%)	
Dead	5 (6%)	9 (9%)	14 (8%)	
Don’t know	16 (20%)	15 (14%)	31 (17%)	
Killed	3 (4%)	10 (10%)	13 (7%)	

*p* values correspond to Chi-square and *t*-test results comparing the intervention and control groups for categorical and numerical variables, respectively.

**Table 3:** Multivariate analysis of the factors associated with compliance with each of the five doses of PEP, and the completion of all the five doses, among bite patients in Makueni County.

	1st Dose Essen Compliance		2nd Dose Essen Compliance		3rd Dose Essen Compliance		4th Dose Compliance		5th Dose Compliance		Completion of All Five Doses	
Parameter	OR	95% CI	OR	95% CI	OR	95% CI	OR	95% CI	OR	95% CI	OR	95% CI
Cost of transport to health facility (USD)	0.999	0.998–1.000										
*Family income per month*												
≤USD 100	Reference category						Reference category					
>USD 100	3.167 *	1.645–6.174					2.382	0.998–6.186				
*Study group*												
Control group			Reference category		Reference category		Reference category		Reference category		Reference category	
Intervention group			4.003 *	1.327–15.314	3.974 *	1.386–14.385	2.708 *	1.091–7.306	6.806 *	2.493–21.521	3.371 *	1.278–10.202
*Category of bite*												
Category 1			Reference category		Reference category		Reference category		Reference category		Reference category	
Category 2			0.513	0.026–3.426	5.228	0.988–29.066	3.635	0.826–16.115	2.262	0.359–10.313	0.296	0.015–1.840
Category 3			3.309	0.149–32.255	9.615 *	1.738–56.816	7.620 *	1.651–36.370	8.393 *	1.293–51.208	0.954	0.047–6.605
*Gender of bite patient*												
Female					Reference category		Reference category				Reference category	
Male					0.270 *	0.092–0.692	0.360 *	0.149–0.821			2.952 *	1.242–7.391
Age of bite patient							0.986	0.966–1.005	0.976 *	0.953–0.998		
*Ownership status of biting dog*												
Known									Reference category			
Unknown									6.512	1.053–58.337		
*Fate of biting animal*												
Alive									Reference category			
Dead									3.411	0.710–25.638		
Unknown									0.414	0.094–1.739		

* *p* value of <0.05.

**Table 4:** Cost of PEP, completion of PEP doses, and reasons for non-completion.

	Treatment Group (*n* = 82)	Control Group (*n* = 104)	Total (*N* = 186)	*p* Value
*Amount spent on PEP (USD)*				0.086
Mean (SD)	3 (9)	1 (5)	2 (7)	
Range	0–39	0–42	0–42	
*Source of money for PEP* **^†^**				0.272
Borrowed	12 (15%)	14 (13%)	26 (14%)	
Loan	3 (4%)	7 (7%)	10 (5%)	
Own savings/income	58 (71%)	82 (79%)	140 (75%)	
Sold an item	18 (22%)	23 (22%)	41 (22%)	
PEP doses completed	76 (93%)	81 (78%)	157 (84%)	0.022
*PEP doses taken on time*				
First dose	25 (31%)	34 (33%)	59 (32%)	0.748
Second dose	76 (95%)	82 (82%)	158 (88%)	0.008
Third dose	71 (91%)	74 (77%)	145 (83%)	0.014
Fourth dose	65 (84%)	67 (74%)	132 (79%)	0.089
Fifth dose	55 (86%)	42 (60%)	97 (72%)	<0.001
*Reason for not completing PEP* **^†^**	*n = 7*	*n = 23*	*n = 30*	0.146
Biting dog was alive	2 (29%)	3 (13%)	5 (17%)	
Forgot	0 (0%)	7 (30%)	7 (23%)	
Lack of funds	2 (29%)	7 (30%)	9 (30%)	
No reason	1 (14%)	1 (4%)	2 (7%)	
PEP not available	3 (43%)	2 (9%)	5 (17%)	
Preoccupied	1 (14%)	3 (13%)	4 (13%)	
Wound healed	0 (0.0%)	1 (4%)	1 (3%)	

**^†^** Households are in more than one category. *p* values correspond to Chi-square and *t*-test results comparing the intervention and control groups for categorical and numerical variables, respectively.

## Data Availability

The data presented in this study are openly available in Open Science Framework: DOI 10.17605/OSF.IO/ZXEQR. This dataset is available under a CC0 1.0 Universal license.

## Supplementary Materials

The following supporting information can be downloaded at https://www.mdpi.com/article/10.3390/vaccines11061112/s1: Supplementary File S1: CONSORT 2010 checklist of information to include when reporting a non-randomized trial; Supplementary File S2: Clinical trial protocol.
