# Supplementary material for: Effect of Phone Text Message Reminders on Compliance with Rabies Post-Exposure Prophylaxis following Dog Bites in Rural Kenya
Source: Vaccines (Basel). 2023 Jun 18;11(6):1112. doi: 10.3390/vaccines11061112 (PMC10303647; doi:10.3390/vaccines11061112)
Supplement: Supplementary file 1 [file vaccines-11-01112-s001.zip › vaccines-2365961-supplementary-conver/Supplementary File S1 CONSORT Checklist.pdf]

CONSORT 2010 checklist of information to include when reporting a non-randomised trial*

Section/Topic	Item No	Checklist item	Reported on page No
**Title and abstract**			
	1a	Identification as a randomised trial in the title<br><br>Title "*Effect of phone text message reminders on compliance with rabies post-exposure prophylaxis following dog-bites in rural Kenya*"<br>*The study is a non-randomized comparative trial where cases/intervention and controls were enrolled into the study based on the time they reported to the health facility. Due to the risky nature of contracting rabies disease once bitten by a rabid dog, all bite patient reporting to the health facility during the intervention period received the text message reminder. The study compared compliance with rabies post exposure prophylaxis (PEP) between the controls- participants that did not receive text reminders and cases (intervention arm) – participants that received text reminders one day prior to their scheduled PEP doses.*	Page 1
	1b	Structured summary of trial design, methods, results, and conclusions (for specific guidance see CONSORT for abstracts)<br>*The abstract has been structured to include context, objectives, trial design and methods, results and conclusion.*	Page 1
**Introduction**			
Background and objectives	2a	Scientific background and explanation of rationale<br>*Explanation of the scientific background and rationale has been explained in the introduction section.*	Page 2
	2b	Specific objectives or hypotheses<br>*The specific objective of the trial is highlighted in the last sentence of the introduction.*	Page 3
**Methods**			
Trial design	3a	Description of trial design (such as parallel, factorial) including allocation ratio.<br>*Information is provided in study design and sample size calculation section.*	Page 3-4
	3b	Important changes to methods after trial commencement (such as eligibility criteria), with reasons<br>*No changes were made to methods after commencement of the study. However, during the phone interview, only participants who responded to the phone interview in the control group were considered for analysis. In the intervention group, all bite patients who responded to the interview and confirmed to have received all four SMS reminders were considered for final data analysis.*	Page 3-4
Participants	4a	Eligibility criteria for participants	

		All study participants reporting to the health facility with a dog bite between October 2018 and March 2019 were enrolled in the study. To be considered in the final analysis, participants who responded to the phone interview in the control group to confirm the number of vaccine doses they had received were considered for analysis. In the intervention group, all bite patients who responded to the interview and confirmed to have received all four SMS reminders were considered for final data analysis.	Page 3-4
	4b	Settings and locations where the data were collected<br>*This information has been provided in the study area section.*	Page 3
Interventions	5	The interventions for each group with sufficient details to allow replication, including how and when they were actually administered.<br>*This information is provided in the study design and sample size calculation section.*	Page 3, 4 and 5
Outcomes	6a	Completely defined pre-specified primary and secondary outcome measures, including how and when they were assessed<br>*Study outcomes are provided in the last paragraph of the study design and sample size calculation section. How and when they outcomes were assessed is stated in the data analysis section.*	Page 5 and 6
	6b	Any changes to trial outcomes after the trial commenced, with reasons<br>*None*	
Sample size	7a	How sample size was determined.<br>*Information is provided in the study design and sample size calculation section.*	Page 5
	7b	When applicable, explanation of any interim analyses and stopping guidelines	
Randomisation:			
Sequence generation	8a	Method used to generate the random allocation sequence<br>*Given the risky nature of contracting rabies disease once bitten by a rabid dog, randomization to either control or intervention group was unethical. Participants were recruited to either control or intervention group based on the time they reported to the health facilities. Bite patients reporting between October -December 2018 were enrolled into the control group. As a routine, this group received a medical card indicating return date for the subsequent dose. All bite patient reporting to the health facility between January and March 2019 were enrolled in the intervention group where they received an SMS reminder a day before the next injection. However, the participants did not know which arm they belonged to.*	
	8b	Type of randomisation; details of any restriction (such as blocking and block size)<br>*NA*	
Allocation concealment mechanism	9	Mechanism used to implement the random allocation sequence (such as sequentially numbered containers), describing any steps taken to conceal the sequence until interventions were assigned - *NA*	

Implementation	10	Who generated the random allocation sequence, who enrolled participants, and who assigned participants to interventions – *The study investigators allocated the participants to either control or intervention group*.	Page 3 and 4
Blinding	11a	If done, who was blinded after assignment to interventions (for example, participants, care providers, those assessing outcomes) and how. *The participants did not know which group they belonged to*.	
	11b	If relevant, description of the similarity of interventions	
Statistical methods	12a	Statistical methods used to compare groups for primary and secondary outcomes *Information available in the data analysis section.*	Page 6
	12b	Methods for additional analyses, such as subgroup analyses and adjusted analyses *Information available in the data analysis section.*	Page 6
**Results**			
Participant flow (a diagram is strongly recommended)	13a	For each group, the numbers of participants who were randomly assigned, received intended treatment, and were analysed for the primary outcome *We have indicated the number of bite patients required for each group. However, we have indicated that all dog bite patients recorded in the register during the study period and in possession of a phone were enrolled in the study to cater for withdrawals.*	Page 4, 6
	13b	For each group, losses and exclusions after randomisation, together with reasons *This information is provided.*	Page 6
Recruitment	14a	Dates defining the periods of recruitment and follow-up *This information is provided in the methodology section, Study Design and Sample Size Calculation.*	Page 4
	14b	Why the trial ended or was stopped *The trial was completed at the expected time at the scheduled time of completion of the five doses of PEP and after conducting the phone interview to collect data on number of doses completed as well as other factors that affecting PEP completion.*	
Baseline data	15	A table showing baseline demographic and clinical characteristics for each group *This information is provided in the result section, Table 1*	Page 6-7
Numbers analysed	16	For each group, number of participants (denominator) included in each analysis and whether the analysis was by original assigned groups *This information is provided*.	Page 6
Outcomes and estimation	17a	For each primary and secondary outcome, results for each group, and the estimated effect size and its precision (such as 95% confidence interval) *This information is provided*.	Page 6-13
	17b	For binary outcomes, presentation of both absolute and relative effect sizes is recommended	

		This information is provided.	Page 6-13
Ancillary analyses	18	Results of any other analyses performed, including subgroup analyses and adjusted analyses, distinguishing pre-specified from exploratory<br>*This information is provided.*	Page 6-13
Harms	19	All important harms or unintended effects in each group (for specific guidance see CONSORT for harms)<br>*NA*	
**Discussion**			
Limitations	20	Trial limitations, addressing sources of potential bias, imprecision, and, if relevant, multiplicity of analyses<br>*Information provided in the last paragraph of the discussion.*	Page 14 and 15
Generalisability	21	Generalisability (external validity, applicability) of the trial findings?<br>*Information is provided in the discussion sections.*	Page 13-14
Interpretation	22	Interpretation consistent with results, balancing benefits and harms, and considering other relevant evidence<br>*Information provided in the discussion section.*	Page 13-14
**Other information**			Page 6
Registration	23	Registration number and name of trial registry<br>*The study trial is registered at US National Institute of Health (clinicalTrial.gov) identifier number NCT05350735*	
Protocol	24	Where the full trial protocol can be accessed, if available<br>*Protocol available online at https://clinicaltrials.gov/ct2/show/NCT05350735*	Page 6
Funding	25	Sources of funding and other support (such as supply of drugs), role of funders<br>*Information provided*	Page 15

*We strongly recommend reading this statement in conjunction with the CONSORT 2010 Explanation and Elaboration for important clarifications on all the items. If relevant, we also recommend reading CONSORT extensions for cluster randomised trials, non-inferiority and equivalence trials, non-pharmacological treatments, herbal interventions, and pragmatic trials. Additional extensions are forthcoming: for those and for up to date references relevant to this checklist, see www.consort-statement.org.
