# Supplementary material for: Effect of Phone Text Message Reminders on Compliance with Rabies Post-Exposure Prophylaxis following Dog Bites in Rural Kenya
Source: Vaccines (Basel). 2023 Jun 18;11(6):1112. doi: 10.3390/vaccines11061112 (PMC10303647; doi:10.3390/vaccines11061112)
Supplement: Supplementary file 1 [file vaccines-11-01112-s001.zip › vaccines-2365961-supplementary-conver/Supplementary File S2 clinical trial protocol.pdf]

ClinicalTrials.gov Protocol Registration and Results System (PRS) Receipt
Release Date: April 21, 2022

ClinicalTrials.gov ID: NCT05350735

Study Identification

Unique Protocol ID: KEMRI/SERU/CGHR/046/3268

Brief Title: Phone Text Message Reminders on Compliance With Human Rabies Post Exposure Prophylaxis Project

Official Title: Effect of Phone Text Message Reminders on Compliance With Rabies Post-Exposure Prophylaxis Following Dog-Bites in Rural Kenya

Secondary IDs:

Study Status

Record Verification: April 2022

Overall Status: Completed

Study Start: October 1, 2018 [Actual]

Primary Completion: August 1, 2019 [Actual]

Study Completion: August 1, 2019 [Actual]

Sponsor/Collaborators

Sponsor: Washington State University

Responsible Party: Sponsor

Collaborators:

Oversight

U.S. FDA-regulated Drug: No

U.S. FDA-regulated Device: No

U.S. FDA IND/IDE: No

Human Subjects Review: Board Status: Approved
Approval Number: KEMRI/SERU/CGHR/046/3268
Board Name: Scientific Ethical Research Unit
Board Affiliation: Kenya Medical Research Institute
Phone: +254 717719477
Email: seru@kemri.org
Address:

KENYA MEDICAL RESEARCH INSTITUTE
SCIENTIFIC AND ETHICS REVIEW UNIT
P.O. BOX 54840 00200 OFF MBAGATHI ROAD,

NAIROBI, KENYA

Data Monitoring:

Study Description

Brief Summary: Every year, rabies, a disease transmitted to humans by rabid dogs, is estimated to kill 59,000 people globally, mostly children 15 years and below in Africa and Asia. This is despite the availability of effective vaccines against rabies in humans, and in dogs. Following a dog bite, there are two critical steps required to prevent clinical disease and death: thorough wound washing with clean running water for at least 15 minutes; and immediate injection with anti-rabies vaccine on the day of being bitten followed by other four injections over the course of one month. Delay in seeking first dose of anti-rabies or failure to complete the recommended dosage may result in clinical rabies and death. The investigators proposed to assess the effect of short message system (SMS) phone text reminders sent to bite patients ahead of their scheduled visits on the adherence to scheduled anti-rabies doses among bite patients in rural eastern Kenya. The investigators enrolled bite patients presenting at Makueni County Referral Hospital between October 2018 and March 2019. Bite patients presenting to the facility between January and March 2019 received SMS reminder written in both English and local dialect a day before each dose of anti-rabies vaccine. These data were compared to those presenting to the health facility in the period prior (October to December 2018) before the introduction of the SMS reminder. This group received routine hospital cards as reminder of their next dose of anti-rabies vaccine injection. Each study participant was contacted after one month from the time of the bite and a phone interview completed to assess compliance and factors associated with completion of the five doses of anti-rabies vaccine.

Detailed Description: Background: Rabies is a fatal viral disease transmitted to humans mainly by domestic dogs. It is a neglected zoonosis that primarily affects underserved populations that have limited access to health care. Every year, rabies is estimated to kill 59,000 people globally, mostly children 15 years and below in Africa and Asia. This is despite the development of effective vaccines against rabies in humans, and in dogs. Although rabies is always fatal once clinical signs manifest, the disease is preventable with timely treatment after exposure to the rabies virus. The World Health Organization (WHO) recommend that bite patients should receive thorough wound cleaning with soap and water for approximately 15 minutes, followed by administration of anti-rabies vaccine on the day of the bite, as well as additional injections over the course of one month. Bite patients with multiple severe bites particularly to the head and upper trunk, infiltration of rabies immunoglobulin into and around the wound is added. Poor availability of rabies vaccines, lack of access to anti-rabies vaccine or deviations from WHO recommendations such as delays in seeking anti-rabies vaccine and incomplete courses of the vaccine increases the risk of clinical rabies and death. Access to mobile phones has increased globally including in rural settings presenting an opportunity to use them to enhance access to health interventions. Text message reminders has been shown to improve patient compliance including for childhood immunization attendance and appointment reminders across different geographical settings and health care services.

Objective: To assess the effect of SMS reminders on compliance with the five-dose Essen rabies vaccine regimen and determine the factors associated with compliance among dog-bite patients.

Methods: The investigators employed a single arm before-after field trial among patients presenting with dog bites in Makueni County Referral Hospital between October 2018 to March 2019. The study participants were allocated to one of

two groups: group 1: bite patients enrolled between January – March 2019 who received a medical card at the first day of visiting the health facility indicating return date for the subsequent dose, and SMS reminders a day before the next dose of anti-rabies vaccine. The SMS messages were written in both English and the local dialect, Kamba; group 2: bite patients recruited into the study between October and December 2018 before the SMS reminder was introduced, this group was designated as the control group. Every bite patient routinely receives a medical card indicating return date for the subsequent dose. To collect data on other factors affecting completion and adherence to the five doses of anti-rabies vaccine, a phone interview was completed to all study participants after at least a month from the bite time.

Conditions

Conditions: Rabies

Keywords: Rabies
SMS
Post exposure prophylaxis
Ant-rabies vaccine
Dog bite patients
Regimen

Study Design

Study Type: Interventional

Primary Purpose: Treatment

Study Phase: N/A

Interventional Study Model: Parallel Assignment

Number of Arms: 2

Masking: Single (Participant)
Participants will not know which arm they belong to.

Allocation: Non-Randomized

Enrollment: 180 [Actual]

Arms and Interventions

Arms	Assigned Interventions
Experimental: Intervention arm: SMS text reminders and medical card<br>Participants in this intervention arm will receive the routine medical card indicating return date for the subsequent doses of anti-rabies vaccine and SMS reminders sent a day before each dose until the scheduled date of the last dose of anti-rabies vaccine.	Behavioral: Intervention arm: SMS text reminders and medical card<br>This study arm will assess compliance with anti-rabies vaccine regimen by participants in the presence of SMS text reminders as compared to without the SMS reminder.
No Intervention: Control arm: Medical card<br>Participants in the control arm will receive the routine medical card indicating return date for the subsequent doses of anti-rabies vaccine. No SMS text reminders will be sent to this group.	

Outcome Measures

Primary Outcome Measure:

1. Number of participants that complete the five-dose Essen rabies vaccine regimen in the control versus intervention group

   The number anti-rabies vaccine doses received by each participant will be reviewed from ant-rabies vaccine register placed at the health facility where participants are recorded after receiving an injection. The investigators will also conduct a phone interview with the participants to determine the number of doses received (using a questionnaire). The investigators will then determine if SMS reminders are associated with completion of the five doses of anti-rabies vaccine.

   [Time Frame: 7 months]

2. Number of participants adhering to the scheduled date of the five-dose Essen rabies vaccine regimen.

   The number of participants adhering to the scheduled date of anti-rabies vaccine doses will be reviewed from ant-rabies vaccine register placed at the health facility where participants are recorded after receiving an injection. The investigators will also conduct a phone interview with the participants to investigate the exact dates participants received the anti-rabies vaccine injections. The investigators will then determine if SMS reminders are associated with adherence to the five doses of anti-rabies vaccine.

   [Time Frame: 7 months]

Eligibility

Minimum Age:

Maximum Age:

Sex: All

Gender Based: No

Accepts Healthy Volunteers: No

Criteria: Inclusion Criteria:

- Participants will be patients of any age reporting to the health facility with dog bites.
- Participants (dog-bite patients or next of kin) in possession of mobile phones.
- Participants (dog-bite patients) consenting to and participating in a phone interview after the last date of the scheduled anti-rabies vaccine dose.
- In the intervention arm - participants acknowledging receipt of SMS text reminders.

Exclusion Criteria:

- Participants (dog-bite patient) that do not consent to participate in the study.
- Participants that do not own mobile phones.
- Participants in the intervention arm that did not receive SMS reminders despite owning mobile phones.

Contacts/Locations

Central Contact Person: Thumbi Mwangi
Telephone: +254 721 250832 Ext. +254
Email: thumbi.mwangi@wsu.edu

Central Contact Backup:

Study Officials: Thumbi Mwangi, PhD

Study Principal Investigator
Washington State University, Paul G Allen School for Global Animal Health

Veronicah M Chuchu, PhD(c)
Study Director
Washington State University - Global Health Kenya

Mutono Nyamai, PhD(c)
Study Director
Washington State University - Global Health Kenya

Locations: **Kenya**
Makueni County Referral Hospital
Makueni, Kenya
Contact: Thumbi Mwangi +254721250832 thumbi.mwangi@wsu.edu
Contact: Veronicah M Chuchu +254716684304 veronicah.chuchu@wsu.edu

IPDSharing

Plan to Share IPD:

References

Citations:

Links:

Available IPD/Information:
